# Potential Roles of IP_3_ Receptors and Calcium in Programmed Cell Death and Implications in Cardiovascular Diseases

**DOI:** 10.3390/biom14101334

**Published:** 2024-10-20

**Authors:** Chanon Piamsiri, Nadezhda Fefelova, Sri Harika Pamarthi, Judith K. Gwathmey, Siriporn C. Chattipakorn, Nipon Chattipakorn, Lai-Hua Xie

**Affiliations:** 1 Department of Cell Biology and Molecular Medicine, Rutgers University-New Jersey Medical School, Newark, NJ 07103, USA; chanon_piam@outlook.co.th (C.P.); fefelonn@njms.rutgers.edu (N.F.);; 2 Cardiac Electrophysiology Research and Training Center, Faculty of Medicine, Chiang Mai University, Chiang Mai 50200, Thailand; 3 Cardiac Electrophysiology Unit, Department of Physiology, Faculty of Medicine, Chiang Mai University, Chiang Mai 50200, Thailand; 4 Center of Excellence in Cardiac Electrophysiology Research, Chiang Mai University, Chiang Mai 50200, Thailand

**Keywords:** IP_3_Rs, programmed cell deaths, cardiovascular diseases, calcium, heart

## Abstract

Inositol 1,4,5-trisphosphate receptors (IP_3_Rs) play a crucial role in maintaining intracellular/cytosolic calcium ion (Ca^2+^_i_) homeostasis. The release of Ca^2+^ from IP_3_Rs serves as a second messenger and a modulatory factor influencing various intracellular and interorganelle communications during both physiological and pathological processes. Accumulating evidence from in vitro, in vivo, and clinical studies supports the notion that the overactivation of IP_3_Rs is linked to the pathogenesis of various cardiac conditions. The overactivation of IP_3_Rs results in the dysregulation of Ca^2+^ concentration ([Ca^2+^]) within cytosolic, mitochondrial, and nucleoplasmic cellular compartments. In cardiovascular pathologies, two isoforms of IP_3_Rs, i.e., IP_3_R1 and IP_3_R2, have been identified. Notably, IP_3_R1 plays a pivotal role in cardiac ischemia and diabetes-induced arrhythmias, while IP_3_R2 is implicated in sepsis-induced cardiomyopathy and cardiac hypertrophy. Furthermore, IP_3_Rs have been reported to be involved in various programmed cell death (PCD) pathways, such as apoptosis, pyroptosis, and ferroptosis underscoring their multifaceted roles in cardiac pathophysiology. Based on these findings, it is evident that exploring potential therapeutic avenues becomes crucial. Both genetic ablation and pharmacological intervention using IP_3_R antagonists have emerged as promising strategies against IP_3_R-related pathologies suggesting their potential therapeutic potency. This review summarizes the roles of IP_3_Rs in cardiac physiology and pathology and establishes a foundational understanding with a particular focus on their involvement in the various PCD pathways within the context of cardiovascular diseases.

## 1. Introduction

Inositol 1,4,5-trisphosphate receptors (IP_3_Rs) are specialized membrane glycoprotein cationic channels responsible for releasing calcium ions (Ca^2+^) from the sarco/endoplasmic reticulum (SR/ER) which is known to be the predominant site of IP_3_Rs localization and serves as the principal site for intracellular Ca^2+^ (Ca^2+^_i_) storage [1,2,3]. The endogenous ligand of IP_3_Rs is inositol 1,4,5-trisphosphate (IP_3_) [1,2]. Upon the interaction between IP_3_ and the inositol binding core of IP_3_Rs, the activation and opening of large-conductance cation channels is initiated [2]. The opening of IP_3_Rs facilitates the release of Ca^2+^ from the SR/ER leading to an elevation in Ca^2+^_i_ concentration ([Ca^2+^]_i_) [2]. There are three isoforms of IP_3_Rs expressed in mammalian cells, namely IP_3_R1, IP_3_R2, and IP_3_R3 [4]. The structural analysis of each IP_3_Rs subtype shows ~70% amino acid sequence homology, despite the fact that all these isoforms originate from three distinct genes [1,5]. All IP_3_R subtypes are essential for regulating Ca^2+^_i_ homeostasis. The binding of IP_3_ to IP_3_Rs plays a crucial role in intracellular communication and in initiating both primary and modulatory signaling [6]. For the primary role, the Ca^2+^ released from IP_3_Rs is used ubiquitously as a secondary messenger to control a variety of intracellular to interorgan physiology and communications including secretion, contraction, protein synthesis, cell division and differentiation, and fertilization [6,7]. The modulatory function of IP_3_R-mediated Ca^2+^ release has been observed in various excitable cell types such as endocrine cells, neurons, and cardiac cells [6]. In these cells, IP_3_Rs modulate the [Ca^2+^]_i_ signal generated by major voltage-operated channels [6].

In addition to their presence in the SR/ER, IP_3_Rs are also distributed in other organelles such as the plasma membrane, Golgi apparatus, mitochondria, and perinuclear/nuclear membranes [8,9,10]. This distribution is observed in various cell types, including neurons and cardiac cells [8,9,10,11]. In the past several decades, a growing body of scientific investigation has demonstrated that IP_3_R signaling participates in different cardiovascular abnormalities [10,12,13,14,15]. More interestingly, a few studies have shown a tight association between IP_3_R signaling and different types of programmed cell death (PCD) [13,14,15,16]. This review article highlights the potential role of IP_3_Rs in various types of PCD, particularly focusing on cardiovascular diseases. In addition, the potential effects of IP_3_R agonists and antagonists are also explored in different disease settings.

## 2. Physiology and Pathology of Calcium Handling in the Heart

**Figure 1:**
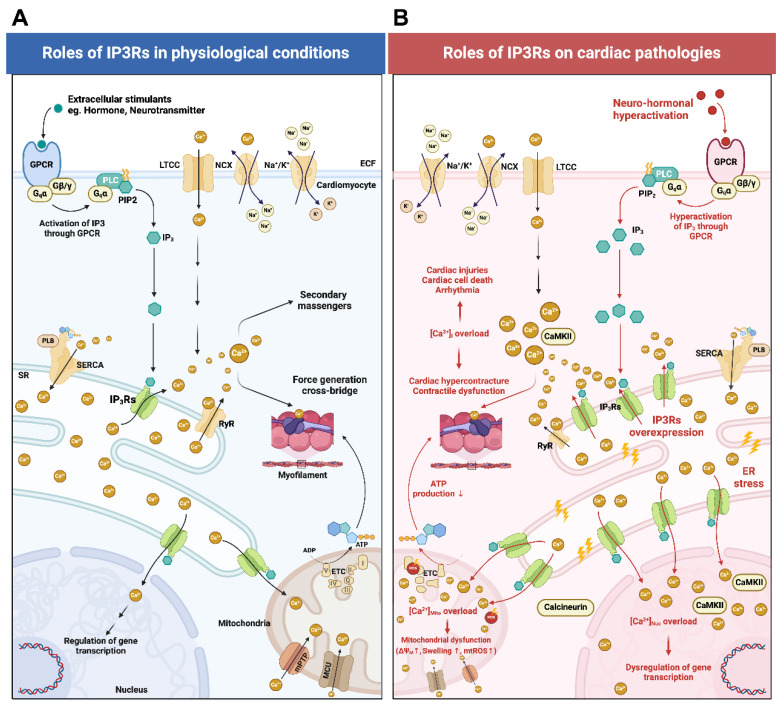
A schematic diagram illustrating the regulatory mechanisms of IP_3_Rs in cardiomyocytes under both physiological and pathological conditions. (**A**) During physiological conditions, external stimuli such as adenosine, angiotensin II, bradykinin, endothelin, vasopressin, dopamine, epinephrine, norepinephrine, etc., bind and activate GPCRs on the plasma membrane. Subsequently, the Gqα-subunit dissociates from the trimeric GPCR structure, activating PLC to produce IP_3_ from PIP2. IP_3_ then binds to the IP_3_ binding core domain of IP_3_Rs leading to the release of Ca^2+^ from the SR. However, the amount of Ca^2+^ flux via IP_3_Rs is relatively lower compared to the Ca^2+^ transients generated by CICR and other major voltage-operated channels and ligand-gated channels. IP_3_R-mediated Ca^2+^ release causes Ca^2+^ translocation from the SR to the cytosol, mitochondria, and nucleus. In the cytosol, Ca^2+^ serves as a secondary messenger playing modulatory roles. Ca^2+^_i_ binds to cTnC and initiates cardiac contraction. In mitochondria, Ca^2+^_Mito_ influences mitochondrial energy production and metabolic activity. Mitochondria also act as secondary storage sites and modulate [Ca^2+^]_i_ levels. In the nucleus, Ca^2+^_Nuc_ regulates gene expression and transcription. (**B**) During cardiac pathologies (e.g., ischemia, I/R injuries, arrhythmias, sepsis-induced cardiomyopathy, cardiac hypertrophy, and heart failure), neurohormonal hyperactivation leads to exacerbated IP_3_ production from GPCR. Additionally, these pathological conditions cause IP_3_Rs overexpression leading to Ca^2+^_i_, Ca^2+^_Mito_, and Ca^2+^_Nuc_ overload. Increased Ca^2+^_i_ binds with calmodulin thus leading to CaMKII overactivation. Ca^2+^_i_ overload causes cardiac hypercontracture, contractile dysfunction, cardiac cell injuries, cardiac cell death, and arrhythmias. Ca^2+^_Mito_ overload causes mitochondrial dysfunction and impaired mitochondrial ATP production. Ca^2+^_Nuc_ overload enhances the function of transcription factors that control the expression of pro-hypertrophic and pro-arrhythmic genes. It should be noted that the remodeling of IP_3_Rs and Ca^2+^ signaling contribute to different cardiovascular diseases and pathophysiological conditions. Endothelin-induced excessive IP_3_R1 expression and overactivation lead to Ca^2+^_i_ and Ca^2+^_Nuc_ overload, subsequently contributing to the development of atrial arrhythmias [6,10,21]. In sepsis-induced cardiomyopathy, the LPS-induced overexpression of IP_3_R2 leads to Ca^2+^_i_ overload triggering myocardial cell death through pyroptosis and apoptosis [14]. In ischemic heart disease, the upregulation of IP_3_R1 in ventricular cardiomyocytes results in Ca^2+^_i_ and Ca^2+^_Mito_ overload leading to myocardial cell death through pyroptosis [6,13]. In the failing heart, persistent increased cardiac workload leads to chronic neurohormonal hyperactivation thereby triggering IP_3_R2/calcineurin/CaMKII-mediated NFAT nuclear translocation, which contributes to the transcription of hypertrophic genes [6,22,23]. This figure was created using BioRender. Abbreviations: ADP, adenosine diphosphate; ATP, adenosine triphosphate; CaMKII, Ca^2+^/calmodulin-dependent protein kinase II; CICR, calcium-induced calcium release; cTnC, cardiac troponin C; GPCR, G protein-coupled receptors; IP_3_, inositol 1,4,5-trisphosphate; IP_3_Rs, inositol 1,4,5-trisphosphate receptors; LTCC, L-type calcium channel; PIP2, phosphatidylinositol 4,5-bisphosphate (PIP2); PLB, phospholamban; PLC, phospholipase C; MCU, mitochondrial calcium uniporter; mPTP, mitochondrial permeability transition pore; mtROS, mitochondrial ROS production; NCX, sodium–calcium exchanger; RyR, ryanodine receptors; SERCA, sarcoplasmic/endoplasmic reticulum Ca^2+^-ATPase; SR, sarcoplasmic reticulum; [Ca^2+^], calcium ion concentration; [Ca^2+^]_i_, calcium ion concentration in the cytosol; [Ca^2+^]_Mito_, calcium ion concentration in mitochondria; [Ca^2+^]_Nuc_ calcium ion concentration in the nucleus; Δ*Ψ_m_* mitochondrial membrane potential.

It is well established that Ca^2+^_i_ plays a critical role in cardiomyocyte excitation–contraction (E-C) coupling [17,18]. Under physiological conditions, proper cardiac contractility is regulated by changes in [Ca^2+^]_i_ during both systole and diastole (Figure 1A). Cardiac contraction during systole is initiated by membrane depolarization leading to the activation of L-type voltage-gated calcium channels (LTCC) located on transverse tubules (T-tubule) [17,19,20]. A cluster of Ca_V_1.2, a predominant cardiac subtype of LTCC, on T-tubules allows trans-sarcolemma Ca^2+^ influx to a cardiac dyad via its concentration gradient and into the cytosol [17,19,20]. Subsequently this change in [Ca^2+^_i_] triggers Ca^2+^-induced Ca^2+^ release (CICR) through the opening of Ca^2+^-dependent ryanodine receptor type-2 (RyR2) on the SR membrane [17,19,20]. As a result, CICR results in an increase in [Ca^2+^]_i_, which interacts with a diverse group of target proteins thereby controlling the biochemical and electromechanical function of the cardiac cell. Ca^2+^_i_ released from the SR binds to cardiac troponin C (cTnC) at the level of the thin myofilaments and initiates cardiac contraction [17]. Therefore, trans-sarcolemma Ca^2+^ influx does not directly cause the contraction of the myofilaments; instead, the myofilaments are activated by the elevation of [Ca^2+^_i_] resulting from Ca^2+^_i_ released from the SR [18]. 

Following membrane depolarization, the LTCC reverts to a slow inactivation state due to its voltage-dependent inactivation (VDI) characteristics [17,20]. Conversely, the resultant Ca^2+^_i_ transient induces the LTCC to enter its fast inactivation state through the Ca^2+^-dependent inactivation (CDI) process [17,20]. This process stops further Ca^2+^ influx from the T-tubules. Under physiological conditions during each cardiac cycle, an equal quantity of Ca^2+^ should be pumped out of the cell as the amount entering [17].

There are two primary mechanisms responsible for lowering Ca^2+^_i_ following E-C coupling. First, Ca^2+^_i_ is efficiently re-sequestered back into the SR via the sarco/endoplasmic reticulum calcium ATPase (SERCA) transporter in an ATP-dependent manner [24]. Indeed, the SR Ca^2+^ re-sequestration by SERCA during diastole is negatively regulated by phospholamban (PLN), which inhibits SERCA during its dephosphorylated state [18,25]. Secondly, the raised Ca^2+^_i_ is pumped out via plasma membrane Ca^2+^-ATPase (PMCA) or exchanged with Na^+^ through the forward mode of the sodium–calcium exchanger (NCX), which serves as the predominant regulation unit for Ca^2+^ efflux in ventricular cardiomyocytes [26,27]. During diastole, about 30% of the Ca^2+^_i_ released from CICR is discharged from the cell via NCX-forward mode and PMCA whereas approximately 70% is resequestered back into the SR by SERCA [28,29].

Interestingly, Ca^2+^_i_ dysregulation is considered one of the pathologic hallmarks across multiple phenotypes of heart failure. In failing myocardium, there are three major components of Ca^2+^_i_ dysregulation: (i) excessive Ca^2+^ entry, (ii) compromised SR Ca^2+^ resequestration, and (iii) exacerbated SR Ca^2+^ leakage [30,31]. These Ca^2+^_i_ dysregulations cumulatively contribute to the impairment of contractility and relaxation of the heart [31,32]. The excessive Ca^2+^ entry is caused predominantly by the hyperphosphorylation of LTCC and the overactivation of the NCX operating in reverse mode (i.e., bringing Ca^2+^_i_ into the cytosol) [29]. The foundation work from Schröder et al. (1998) reported that the channel availability and the open probability of LTCC were importantly higher in cardiomyocytes isolated from failing human hearts compared to samples from non-failing human hearts [33]. They also reported the augmented steady-state level of LTCC phosphorylation in failing human myocardium [33] which allowed excessive Ca^2+^ entry [29]. The recent work of Sanchez-Alonso and colleagues further clarified that the increase in the open probability of LTCC is regulated by the loss of coupling regulation of β-2 adrenergic receptors during heart failure [34]. In addition, the upregulation of NCX in the sarcolemma combined with the NCX operating in the reverse mode during heart failure magnifies the impact of an elevation in intracellular sodium concentration ([Na^+^]_i_) [35]. The elevation of [Na^+^]_i_ might be partially caused by an elevated late Na^+^ current, diastolic Na^+^ influx, and/or a reduced sodium–potassium pump function, which activates the reverse mode of the NCX in human cardiomyocytes during heart failure [36,37]. The resulting NCX-reverse mode overactivation facilitates Ca^2+^ influx and impairs Ca^2+^ discharge during diastole, culminating in Ca^2+^_i_ overload and subsequent diastolic dysfunction [31,38].

In addition, the store-operated calcium entry (SOCE) mechanism has also been reported to enhance Ca^2+^ influx during human heart failure [39,40,41]. Previous studies have reported a reduction in SERCA expression [42] alongside a decrease in PLN phosphorylation [43] resulting in compromised SR Ca^2+^ resequestration by SERCA in human failing myocardium. Unlike SERCA, the expression of RyR during heart failure is not changed [44]. Rather, increased β-adrenergic signaling increases adenylyl cyclase (AC) cyclic adenosine monophosphate (cAMP) production and cAMP-dependent protein kinase (PKA) phosphorylation facilitates RyR hyperphosphorylation at Ser 2809 [45,46,47]. The resultant RyR hyperphosphorylation leads to channel instability, dysfunction, and Ca^2+^ leakage ultimately resulting in diastolic SR Ca^2+^ leakage during heart failure [45,46,47]. The pathological SR Ca^2+^ leakage not only impacts RyR hyperphosphorylation, but also triggers Ca^2+^/calmodulin-stimulated protein kinase II (CaMKII) which subsequently phosphorylates LTCC exacerbating excessive Ca^2+^ entry [48]. Taken together, the optimal and tightly controlled regulation of [Ca^2+^]_i_ is essential for the effective electromechanical coupling of the heart during both systole and diastole. The dysregulation of Ca^2+^_i_ and/or the alteration in Ca^2+^ modulation proteins in the heart can inevitably compromise both contraction and relaxation ultimately leading to heart failure.

## 3. Physiology of IP_3_R-Mediated Ca^2+^ Signaling

In the heart, Ca^2+^_i_ is known as a crucial regulator of intracellular signaling, arrhythmogenesis, gene transcription, and controlling cardiac contractility [12,49]. It has been recognized that the SR is also the primary site for harboring IP_3_Rs in addition to RyRs, both of which regulate the release of Ca^2+^ from the SR (Figure 1A). Both types of channels share many similar features and functions. Although the Ca^2+^ conductance of IP_3_Rs is restricted to IP_3_ engagement, both RyRs and IP_3_Rs display a biphasic response to changes in [Ca^2+^] in the lumen of the SR [50]. At lower concentrations, Ca^2+^ facilitates channel conduction, whereas at higher concentrations it induces channel inactivation [50]. In general, it has been demonstrated that IP_3_ and IP_3_Rs serve as precise modulators that finely tune the Ca^2+^_i_ signals produced by major voltage-operated channels [6]. The process in which IP_3_ interacts with IP_3_Rs leading to the release of Ca^2+^ is termed IP_3_-induced Ca^2+^ release or IICR [51]. Although the amount of Ca^2+^ flux via IP_3_Rs is relatively lower in comparison to the Ca^2+^ transients generated by major voltage-operated channels or ligand-gated channels [12] and SR Ca^2+^ release, the IP_3_Rs are large-conductance cationic channels that are found localized in the ER/SR, plasma membrane, Golgi apparatus, mitochondria, and perinuclear/nuclear membrane of cardiomyocytes [2,6,10]. The subcellular distribution, spatial relationships, and functional roles of IP_3_Rs responsible for the release of Ca^2+^ from the ER/SR lumen into the cytosol and associated organelles have been studied [6,52]. The primary function of IP_3_Rs in cardiac cells is to amplify the frequency and amplitude of Ca^2+^ sparks and to sensitize Ca^2+^ oscillations triggered by major voltage-operated channels, thereby facilitating myocardial contraction [6]. In cardiac cells including atrial and ventricular cardiomyocytes as well as in Purkinje cells, IP_3_Rs are located at the SR adjacent to the T-tubule, Z-lines, mitochondria-associated SR membranes, and perinuclear region [2,8,53,54,55,56,57]. Additionally, it has been demonstrated that the presence of IP_3_Rs is not only necessary for ER–mitochondrial Ca^2+^ propagation, but also crucial for maintaining physical ER–mitochondrial contact [2]. The formation of IP3Rs into a channel complex with mitochondrial-associated proteins (i.e., GRP75, VDAC, and MCU) at the mitochondria-associated ER membranes is responsible for mitochondrial Ca^2+^ uptake and ER–mitochondrial oxidative stress in both cardiac and non-cardiac cells [58,59,60]. In atrial cardiomyocytes, the activation of IP_3_Rs on the nuclear envelope is responsible for the modification of arrhythmic gene transcription through calcineurin and CaMKII which promotes class-II histone deacetylase contributing to cardiac remodeling and arrhythmias [10,54]. In ventricular cardiomyocytes, the activation of nucleoplasmic IP_3_Rs triggers calcineurin/CaMKII signaling leading to the nuclear translocation of the nuclear factor of activated T cells (NFAT), which contributes to hypertrophic remodeling [8].

The expression of all three IP_3_Rs isoforms have been reported in cardiomyocytes [61,62]. However, previous studies have reported that among the three IP_3_Rs subtypes, IP_3_R2 appears to be the predominant isoform expressed in adult cardiomyocytes being approximately six times more prevalent in the atria compared to the ventricles [14,61,62,63]. In the fetal heart, IP_3_R1 expression is detected predominantly, albeit at substantially lower levels compared to the predominant expression of IP_3_R2 in adult atria [22]. IP_3_R1 has also been found in Purkinje myocyte [8,53]. Meanwhile, IP_3_R3 is nearly absent in adult myocardium [22]; rather, it plays a crucial role in cardiomyocyte differentiation during embryogenesis [64,65]. The structure of all IP_3_R isoforms comprises a tetrameric complex consisting of five domains: the IP_3_ binding core domain, the central regulatory domain, the suppressor domain, the transmembrane domain, and the *C*-terminus domain [66]. IP_3_Rs are activated through the selective binding of IP_3_ to an allosteric site at the IP_3_ binding core (IBC) domain [66]. This interaction triggers the channel opening of IP_3_Rs by facilitating the binding of Ca^2+^ to a stimulatory Ca^2+^-binding site ultimately resulting in Ca^2+^ efflux from storage sites [67]. The release of Ca^2+^ from IP_3_Rs activates Ca^2+^-sensitive adenylyl cyclase (i.e., AC1 and AC8), consequently enhancing the cAMP/PKA pathway, thus strengthening CICR mediated by the RyR [22]. In addition, Ca^2+^ released from IP_3_Rs also facilitates the distribution of Ca^2+^ into mitochondria and nuclei, thereby influencing and regulating mitochondrial bioenergetics and nuclear gene transcription [2,10,22]. However, in the failing heart, the heightened Ca^2+^_i_ released from RyR caused by IP_3_Rs also exacerbates SR Ca^2+^ leakage while impairing mitochondria Ca^2+^ ([Ca^2+^]_Mito_) sequestration [22,68]. If the resultant SR Ca^2+^ leak reaches sufficient magnitude, it can enhance the activity of sarcolemmal NCX inducing membrane depolarization that can result in delayed afterdepolarization (DAD), and potentially amplify the generation of triggered action potentials (or triggered activities) [22,68]. Also, it has been reported that Ca^2+^_i_ transients in tissues from patients with heart failure are markedly prolonged, which is related to depleted SR Ca^2+^ re-sequestration during diastole [18,30,69]. These cumulative effects amplify disruptions in myocardial Ca^2+^ homeostasis potentially contributing to compromised cardiac function. Recently reported to be associated with the pathogenesis of several cardiac and non-cardiac diseases, IP_3_R-mediated Ca^2+^ flux has been shown to contribute to an augmentation of intracellular and intra-organelle [Ca^2+^]. In the subsequent section, we review and discuss the remodeling of IP_3_Rs and their intricate roles in cardiac pathologies. A schematic diagram summarizing the regulatory mechanism of IP_3_Rs in cardiovascular diseases is illustrated in Figure 1B.

## 4. Remodeling of IP_3_Rs and Roles in Cardiac Pathologies

IP_3_Rs are more highly expressed in the heart during embryogenesis compared to during the adult stage [70]. Although IP_3_R2 has been reported to be the predominant isoform in adult cardiomyocytes, the genetic ablation of IP_3_R2 (i.e., homozygous IP_3_R2^−/−^ mice) did not alter embryonic development, animal fertility, or overall systemic physiological functions [21]. In contrast, the selective overexpression of cardiac IP_3_R2 was observed to be associated with cardiac hypertrophy and the occurrence of increased arrhythmias [23,71]. Meanwhile, homozygous IP_3_R1^−/−^ mice were found to have embryonic fatality and post-developmental abnormalities [21]. These findings suggest that in physiological versus pathological conditions, IP_3_Rs may play distinct roles indicating a nuanced interplay between their functions in different contexts. In line with this, several studies have reported that the elevation of IP_3_ and IP_3_Rs in adult cardiomyocytes is associated with the development of various cardiac abnormalities including ischemia, ischemic/reperfusion (I/R) injury, arrhythmias, sepsis-induced cardiomyopathy, cardiac hypertrophy, and heart failure. Gomez and colleagues reported that IP_3_R1 which is phosphorylated at Ser1756 (p-_Ser1756_ IP_3_R1) and glycogen synthase kinase-3β (GSK3β) were both increased in cultured cardiomyoblasts (H9c2 cells) under hypoxic–reoxygenation (H/R) insults resulting in mitochondrial Ca^2+^ (Ca^2+^_Mito_) overload and cell death [72]. The elevation of IP_3_R1 and p-_Ser1756_ IP_3_R1 protein expression were also observed in cardiac tissue after I/R in mice [72]. In this study, they reported that GSK3β specifically interacted with IP_3_Rs as a channel complex at the mitochondria-associated ER membranes [72]. Treatment with the GSK3β inhibitor SB216763 mitigated the increase in IP_3_R1 and p-_Ser1756_ IP_3_R1 protein expression and ER-mediated Ca^2+^_i_ and Ca^2+^_Mito_ overload in the I/R mouse hearts [72]. These results suggest that cardiac I/R mediates the overexpression of IP_3_R1, p-_Ser1756_ IP_3_R1, and GSK3β. These proteins have been shown to have an interaction as a channel complex responsible for Ca^2+^ flux from the ER to mitochondria resulting in Ca^2+^_Mito_ overload and cardiac cell death. In line with these finding, Mo et al. observed a consistent upregulation of IP_3_R1 in cultured primary rats cardiomyocytes and H9c2 cells subjected to hypoxia/reperfusion (H/R) conditions [13]. Additionally, this upregulation was also noted in cardiac tissue in rats experiencing I/R [13]. In addition, the downregulation of endoplasmic reticulum resident protein 44 (ERP44) which is known to participate in Ca^2+^ homeostasis and to modulate IP_3_R1 was also observed [13]. In contrast, genetic modifications such as IP_3_R1 ablation and ERP44 overexpression were found to limit Ca^2+^_i_ and Ca^2+^_Mito_ overload and to reduce infarct size and cardiac injury, as well as to reduce cardiac cell death, among rats subjected to I/R insult and in cells exposed to H/R [13]. Taken together, upon H/R and I/R, there was an upregulation of IP_3_R1, p-_Ser1756_ IP_3_R1, and GSK3β and a downregulation of ERP44 leading to Ca^2+^_i_ and Ca^2+^_Mito_ overload and cardiac cell death. Both the pharmacological modulation and the genetic modification of IP_3_R1 and its related proteins have shown promising therapeutic potential by mitigating IP_3_R1-mediated Ca^2+^_i_ and Ca^2+^_Mito_ overload and improving cardiac cell survival in I/R-related pathological conditions. Additionally, a study from Santullia and colleagues demonstrated that IP_3_R2 levels were upregulated at both the mRNA and protein levels and were accompanied by changes in Ca^2+^ handling in the cytoplasm and mitochondria [73]. There was also increased mitochondrial oxidative stress after non-reperfused myocardial infarction [73]. However, the genetic ablation of IP_3_R2 did not impact Ca^2+^ spark frequency, SR Ca^2+^ load, Ca^2+^_Mito_ overload, or mitochondrial oxidative stress caused by non-reperfused myocardial infarction [73]. Collectively, these data suggest that IP_3_R2 may not contribute to mitochondrial dysfunction in the context of non-reperfused myocardial infarction [74]. Nevertheless, it is important to note that the expression of other IP_3_R isoforms (i.e., IP_3_R1 and IP_3_R3) were not assessed in this study [73].

In addition to cardiac ischemia, previous studies have also addressed the essential roles of IP_3_Rs in relation to arrhythmogenesis when exposed to high glucose concentrations and during diabetes-related conditions. Yuan and colleagues demonstrated a significant increase in IP_3_R1 and its corresponding protein glucose-regulated protein 75 (GRP75) expression in isolated primary rat atrial cardiomyocytes cultured under high glucose conditions [15]. They observed an increase in both [Ca^2+^]_i_ and [Ca^2+^]_Mito_ compared to the control (normal glucose) group [15]. Their recent work in 2022 also assessed the marked increase in IP_3_R1 both in the cultured HL-1 cell line (mouse atrial cardiomyocytes) under high glucose conditions and in rats with type II diabetes [60]. More interestingly, the in situ proximity ligation results revealed that IP_3_R1 and GRP75 have interactions with voltage-dependent anion channels (VDAC) as an IP_3_R1-GRP75-VDAC complex [60]. The increase in IP_3_R1 expression and its complex formation was responsible for ER stress, which elevated [Ca^2+^]_i_ and [Ca^2+^]_Mito_ in the diabetic rats [60]. Consequently, the IP_3_R1-mediated Ca^2+^_i_ and Ca^2+^_Mito_ overload were found to be associated with mitochondrial dysfunction, adverse atrial remodeling, and increased atrial fibrillation (AF) inducibility in diabetic rats [60]. Furthermore, the genetic ablation of the protein GRP75 was found to effectively attenuate atrial remodeling and arrhythmias in type II diabetic rats [60]. Collectively, high glucose and diabetic conditions significantly augmented the expression of IP_3_R1 and IP_3_R1-related protein GRP75 leading to ER–mitochondrial channel complex formation. This ultimately caused Ca^2+^_i_ and Ca^2+^_Mito_ overload, mitochondrial dysfunction, atrial remodeling, and AFs. Genetic modification of the IP_3_R1-related protein GRP75 showed promising beneficial effects against diabetes-induced AFs.

One of the underlying mechanisms for AF has been attributed to abnormalities in atrial cardiomyocyte Ca^2+^_i_, such as spontaneous SR Ca^2+^ release, which augments [Ca^2+^]_i_ [75]. Li and colleagues reported that the global ablation of IP_3_R2 abolished the increase in diastolic [Ca^2+^]_i_ and Ca^2+^_i_ transient amplitude in atrial cardiomyocytes to a similar extent as the IP_3_R antagonist 2-aminoethoxy-diphenyl borate (2-APB) in endothelin-1-stimulated mice [21]. In a previous study, Qi et al. elucidated the underlying mechanisms by which AF not only altered Ca^2+^_i_ handling, but also affected nuclear Ca^2+^ concentration ([Ca^2+^]_Nuc_) through the involvement of IP_3_R1 [10]. In this study, they isolated atrial cardiomyocytes from the atria of tachycardia-paced dogs. The results showed that these AF dogs had upregulation of IP_3_R1 in both the nucleoplasmic and cytoplasmic fraction, while an increase in IP_3_R2 was observed only in the nucleoplasmic fraction [10]. In this study, diastolic [Ca^2+^] was determined during the depolarization phase under 1Hz stimulation followed by a 10 s pause to assess [Ca^2+^] during the resting stage. The isolated dog atrial cardiomyocytes with AF were found to have significantly higher [Ca^2+^]_i_ and [Ca^2+^]_Nuc_ levels during both the diastolic and the resting phase when compared to the control group [10]. The administration of IP_3_, the endogenous ligand of IP_3_Rs, further increased [Ca^2+^]_i_ and [Ca^2+^]_Nuc_ in isolated atrial cardiomyocytes from tachycardia-paced dogs [10]. Indeed, only IP_3_R1 knockdown could mitigate diastolic [Ca^2+^]_Nuc_ alterations. This cardioprotective effect was not observed when IP_3_R2 was knocked down [10]. Furthermore, treatment with the IP_3_R antagonist 2-APB prevented IP_3_-mediated increase in Ca^2+^_i_ and Ca^2+^_Nuc_ overload [10]. These findings suggest that AF-associated [Ca^2+^]_Nuc_ alterations are likely via IP_3_R1, but not IP_3_R2. IP_3_R1 plays a predominant role in regulating the [Ca^2+^]_Nuc_ in atrial cardiomyocytes isolated from AF dogs and in vitro tachy-paced atrial cardiomyocytes.

Very recently, Wu and colleagues studied sepsis-induced cardiomyopathy by injecting lipopolysaccharide (LPS) into rats. They found that LPS administration aggravated ER stress and increased the expression of IP_3_R2 resulting in Ca^2+^_i_ overload and cardiac cell death [14]. In this study, treatment with ER stress antagonist 4-phenylbutyric acid (4-PBA), genetic ablation of IP_3_R2, or pharmacological inhibition of IP_3_R2 using Xestospongin C reversed LPS-induced cardiomyocyte Ca^2+^_i_ overload [14]. Hence, this study highlights the role of IP_3_R2 in being responsible for LPS-induced cardiomyocyte Ca^2+^_i_ overload. 

Moreover, there is also evidence implicating IP_3_R2 in cardiac hypertrophy. The previous work by Roderick et al. observed that IP_3_R2 was upregulated in spontaneously hypertensive rats (SHRs), aortic banded mice, and in heart failure patients with cardiac hypertrophy and secondary ischemic dilated cardiomyopathy [12]. Their SHR strain, known for spontaneous cardiomyocyte hypertrophy, showed higher systolic Ca^2+^_i_ transient amplitudes. There was a tendency for the left ventricular fraction shortening (%LVFS) to be lower compared to wildtype mice under basal conditions, although this difference was not statistically significant [12]. Stimulation with IP_3_ promoted greater Ca^2+^_i_ transient amplitudes and increased LVFS among the SHR strain more than in the wildtype strain. These effects were abolished by the IP_3_R antagonist 2-APB [12]. In addition to elucidating the roles of IP_3_R2 in cardiac hypertrophy, their findings also underscored the effectiveness of an IP_3_R antagonist in mitigating alterations in Ca^2+^_i_ transients induced by IP_3_ stimulation. Their later work in 2012 also reported a significant increase in IP_3_R2 protein in the ventricular tissue of aortic banded mice [76]. These mice exhibited higher ventricular weight, elevated atrial natriuretic peptide levels, and lower LVFS indicating pressure overload-induced cardiac hypertrophy and heart failure [76]. The expression levels of miRNA-133a, known for its anti-hypertrophic properties, were also decreased in the aortic banded mice [76]. The researchers also performed miRNA-133a double knockout in mice and found that IP_3_R2 protein levels were increased, while the protein expression of IP_3_R1 and IP_3_R3 was not affected [76]. Conversely, the overexpression of miR-133a mitigated IP_3_R2 protein expression [76]. Taken together, miRNA-133a negatively regulated IP_3_R2 expression, Ca^2+^ signals, and cardiac hypertrophy. Reports from in vitro, in vivo, and clinical studies support the concept that the overactivation of IP_3_Rs is associated with the pathogenesis of various cardiac pathologies through the dysregulation of Ca^2+^ handling in cytosolic, mitochondrial, and nucleoplasmic compartments. According to previous reports mentioned above, it is evident that IP_3_R1 and IP_3_R2 exhibit distinct involvement in different cardiovascular abnormalities. IP_3_R1 emerges as playing a pivotal role in the development of cardiac ischemic injury and arrhythmias, while IP_3_R2 is implicated in sepsis-induced cardiomyopathy and cardiac hypertrophy. Both genetic modification and pharmacological intervention targeted on IP_3_Rs and their associated proteins exhibit promising therapeutic efficacy in alleviating pathologies linked to IP_3_Rs.

## 5. The Roles of IP_3_Rs and Ca^2+^ Signaling in Different Types of Programmed Cell Death

### 5.1. IP_3_Rs, Ca^2+^ Signaling, and Apoptosis (Figure 2A)

PCD in the heart is a fundamental process that occurs in both physiological and pathological conditions. This process is intricately ‘programmed’ by various predominant signaling pathways including apoptosis, pyroptosis, necroptosis, and ferroptosis [77,78]. Apoptosis is a form of programmed cell death (PCD) that plays a crucial role in various cardiovascular abnormalities including atherosclerosis, diabetic cardiomyopathy, cardiac arrhythmias, ischemic heart diseases, and heart failure [78,79]. The major mechanisms of apoptosis consist of extrinsic (death receptor dependent) and intrinsic (mitochondrial dependent) pathways [80]. The extrinsic apoptosis pathway is activated by ligand binding and the activation of transmembrane receptors such as tumor necrosis factor receptor 1 (TNFR1), toll-like receptors (TLRs), and FAS receptors (FASR) [81]. The interaction of these transmembrane receptors with their corresponding ligands (e.g., TNF-α for TNFR1, LPS for TLR4, and FAS ligand for FASR) activate downstream signaling molecules. This activation eventually leads to proteolytic caspase-mediated processes including cell shrinkage, chromosomal condensation, DNA fragmentation, and ultimately cell death [77,78,79,80,81]. In contrast to extrinsic apoptosis, internal stressors such as oxidative stress, osmotic stress, proteotoxic stress, nutritional stress, or DNA damage can trigger mitochondrial stress and injury [77,78,79,80,81]. This convergence leads to the opening of mitochondrial permeability transition pores (mPTP) followed by the release of cytochrome c from the mitochondrial intermembrane space into the cytoplasm [77,78,79,80,81]. The presence of intracytoplasmic cytochrome c facilitates the assembly of the apoptosome, which in turn activates downstream caspases, ultimately initiating apoptotic execution [77,78,79,80,81]. Substantial evidence has demonstrated that apoptosis contributes to the progression of heart failure by causing the ongoing loss of functional myocardium leading to adverse clinical outcomes [82,83]. It has been reported that evidence of apoptosis phenotypes (i.e., DNA fragmentation and caspase 3) can be identified in the myocardial tissue obtained from patients suffering from conditions such as myocarditis, dilated cardiomyopathy, diabetic cardiomyopathy, myocardial infraction, and end-stage congestive heart failure [79,84,85]. 

**Figure 2:**
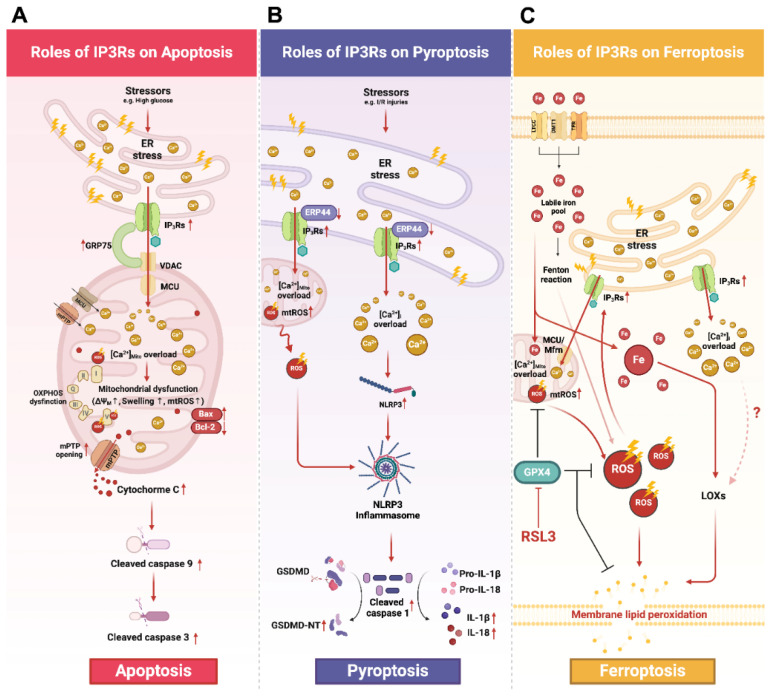
A schematic diagram illustrating the roles of IP_3_Rs in apoptosis, pyroptosis, and ferroptosis. (**A**) Apoptosis: Several stress conditions (e.g., high glucose condition) mediate ER stress resulting in the upregulation of IP_3_Rs and GRP75 expression. Formation of the IP_3_Rs-GRP75-VDAC complex facilitates ER-mediated Ca^2+^_Mito_ overload. Excessive [Ca^2+^]_Mito_ causes mitochondrial dysfunction and facilitates mPTP opening, leading to Cytochrome C being released. The access of Cytochrome C to the cytosol initiates mitochondrial-mediated apoptosis by activating caspase cascades, including the cleavage of caspase 9 and caspase 3. (**B**) Pyroptosis: Stressors such as I/R or H/R induce the overexpression of IP_3_Rs and the downregulation of ERP44, thereby mediating Ca^2+^_Mito_ and Ca^2+^_i_ overload. Ca^2+^_Mito_ overload impairs mitochondrial functions, disrupts oxidative phosphorylation, and increases mtROS production. The resultant overproduction of mtROS and Ca^2+^_i_ overload lead to NLRP3 activation and inflammasome assembly. Once activated, the NLRP3 inflammasome cleaves pro-caspase 1 to its activated form. Then, cleaved caspase 1 further cleaves and activates GSDMD into its *N*-terminal fragment (GSDMD-NT). As a result, GSDMD-NT translocates and oligomerizes at the inner leaflet of the plasma membrane resulting in the formation of transmembrane pores. (**C**) Ferroptosis: It is proposed that ER stress could mediate ferroptosis through the overactivation of IP_3_Rs, thereby, leading to Ca^2+^_Mito_ overload and mtROS overproduction. The administration of ferroptosis activator RSL3 inhibited the antioxidant property of GPX4 exacerbating mitochondrial and intracellular ROS levels. The subsequent augmentation of ROS leads to membrane lipid peroxidation and ultimately causes membrane rupture. In addition, Ca^2+^_i_ overload caused by IP_3_Rs also enhances the catalytic activity of LOX, further promoting lipid membrane peroxidation. However, the underlying mechanism of whether and how Ca^2+^_i_ impacts LOX remains unclear. This figure was created using BioRender. Abbreviations: BAX, Bcl-2-associated protein X; Bcl-2, B-cell lymphoma 2; GSDMD, gasdermin D; GSDMD-NT, gasdermin D *N*-terminus; H/R, hypoxia-reoxygenation; IP_3_Rs, inositol 1,4,5-trisphosphate receptors; I/R, ischemia–reperfusion; LOX, lipoxygenases; MCU, mitochondrial calcium uniporter; MFRN, mitoferrin 2; mPTP, mitochondrial permeability transition pore; mtROS, mitochondrial ROS; NLRP3, nod-like receptor protein-3; RSL3, RAS-selective lethal 3; [Ca^2+^], calcium ion concentration; VDAC, voltage-dependent anion channel; [Ca^2+^]_i_, calcium ion concentration in the cytosol; [Ca^2+^]_Mito_, calcium ion concentration in mitochondria; [Ca^2+^]_Nuc_ calcium ion concentration in the nucleus; Δ*Ψ_m_*, mitochondrial membrane potential.

Recently, Ca^2+^ signaling and its related proteins have been extensively demonstrated to play critical roles in the development of different types of PCD pathways [80,86,87]. However, there have been limited investigations into the roles of IP_3_Rs in various types of PCD and even fewer studies have addressed their role in the context of cardiovascular abnormalities. As previously mentioned, high glucose conditions were found to induce an upregulation of IP_3_R1 and its associated protein GRP75 accompanied by Ca^2+^_Mito_ overload and subsequent cardiac mitochondrial dysfunction in cultured HL-1 cells [15,60]. It is well noted that Ca^2+^_Mito_ overload can ultimately induce mitochondrial dysfunction [88,89]. This process, in turn, triggers the opening of mPTP ultimately leading to mitochondrial-dependent apoptosis [88,89]. Consistently, there is also a concurrent increase in the ratio of Bcl-2-associated protein X (BAX) to B-cell lymphoma 2 (Bcl-2) and annexin V-positive cells indicating the activation of apoptosis in HL-1 cells under high glucose conditions [15,60]. Notably, the genetic ablation of GRP75 has been shown to mitigate the upregulation of pro-apoptotic proteins including cleaved caspase 9/pro-caspase 9, cleaved caspase 3/pro-caspase 3, and the BAX/Bcl-2 ratio in atrial cardiomyocytes under ER stress conditions [60]. Moreover, GRP75 silencing has also been shown to reduce the distance between the ER and the mitochondrial outer membrane, to improve [Ca^2+^]_Mito_ levels, and to alleviate cardiac mitochondrial dysfunction [60]. Taken together, these finding indicate that under high glucose conditions, the disruption of ER–mitochondria Ca^2+^ handling occurs via the interaction of the IP_3_R1-GRP75-VDAC complex. This disruption mediates ER stress, Ca^2+^_Mito_ overload, mitochondrial dysfunction, and subsequent apoptosis. Therefore, genetic ablation of the IP_3_R1-related protein GRP75 effectively attenuates high-glucose-induced apoptosis in atrial cardiomyocytes by mitigating ER–mitochondria interactions.

### 5.2. IP_3_Rs, Ca^2+^ Signaling, and Pyroptosis (Figure 2B)

Pyroptosis is a type of PCD that primarily arises from the caspase-1-dependent generation of transmembrane pores leading to the release of pro-inflammatory cytokines and cell lysis [90,91]. An increasing body of evidence indicates that pyroptosis plays a significant role in various cardiovascular abnormalities such as cardiac I/R injury, myocardial infarction, and heart failure [92,93,94,95]. In general, pyroptosis is initiated by the interaction between pathogen-associated molecular patterns (PAMPs) and damage-associated molecular patterns (DAMPs) with pattern recognition receptors (PRRs), which leads to the activation of inflammasomes [80,96]. The activation and assembly of inflammasomes lead to the activation of proteolytic caspase 1, which then cleaves gasdermin D (GSDMD) into the gasdermin D *C*-terminus (GSDMD-CT) and *N*-terminus (GSDMD-NT) [80,96]. As a result, GSDMD-NT translocates and oligomerizes at the inner leaflet of the plasma membrane leading to the formation of transmembrane pores and cell lysis [80,96,97]. Apart from an external stressor, several internal stimuli have been identified as contributors to inflammasome-mediated pyroptosis. It has been reported that the assembly of inflammasomes and the occurrence of pyroptosis are facilitated by an increase in mitochondrial reactive oxygen species (mtROS) and the release of mitochondrial DNA (mtDNA) from impaired and dysfunctional mitochondria [94,95]. 

In addition, a study from Mo et al. also reported the involvement of IP_3_R1 in Ca^2+^ transport and pyroptosis in a cardiac I/R model [13]. In this study, they reported the upregulation of IP_3_R1 and downregulation of IP_3_R1 modulator ERP44 in cardiac tissue from rats exposed to I/R [13]. They also noted a resultant Ca^2+^_i_ and Ca^2+^_Mito_ overload [13]. The upregulation of pyroptosis markers, including caspase 1, nod-like receptor protein-3 (NLRP3), apoptosis-associated speck-like protein containing a CARD, caspase recruitment domain (ASC), GSDMD-NT, interleukin 1β (IL-1β), and interleukin 18 (IL-18) were also observed in both I/R rats and H/R cells [13]. Notably, IP_3_R1 silencing and ERP44 overexpression effectively alleviated IP_3_R1-mediated Ca^2+^_i_ and Ca^2+^_Mito_ overload leading to a reduction in pyroptosis-pertinent proteins in rats subjected to ischemia/reperfusion (I/R) [13]. Taken together, the perturbation of Ca^2+^_i_ is commonly recognized as one of the prerequisites for NLRP3 activation and subsequent pyroptosis [98,99,100]. These results indicated that cardiac I/R gave rise to IP_3_R1 and ERP44 overexpression, thus leading to Ca^2+^_i_ and Ca^2+^_Mito_ overload and subsequently myocardial pyroptosis. Notably, genetic manipulation involving IP_3_R1 silencing and ERP44 overexpression have been shown to potentially mitigate IP_3_R1-mediated pyroptosis under I/R conditions.

Furthermore, Wu et al. highlighted that LPS administration caused IP_3_R2-mediated Ca^2+^_i_ overload, triggering NLRP3-driven pyroptosis, and cardiac dysfunction in Sprague Dawley rats administered LPS [14]. In this study, the intraperitoneal administration of LPS augmented IP_3_R2 expression, which activated the formation of NLRP3 inflammasomes and elevated pyroptosis markers (i.e., IL-1β, IL-18, cleaved caspase 1, and GSDMD-NT) in the left ventricle leading to contractile dysfunction [14]. In vitro experiments also confirmed that LPS treatment induced IP_3_R2 overexpression and Ca^2+^_i_ overload in isolated neonatal rat atrial cardiomyocytes (NRCMs) [14]. Silencing IP_3_R2 effectively reversed LPS-induced Ca^2+^_i_ overload in NRCM cardiomyocytes [14]. Additionally, either the genetic ablation of IP_3_R2 or the pharmacological inhibition of IP_3_R2 significantly reduced pyroptosis activation in NRCMs under LPS stimulation [14]. In addition to IP_3_R2 overactivation, the upregulation of ER stress markers was also observed, including the activation of transcription factor 4 (ATF4) and CCAAT-enhancer-binding protein homologous protein (CHOP) in both myocardial tissues and NRCM cells following LPS stimulation [14]. These insults could be mitigated by the ER stress antagonist 4-BPA [14]. Collectively, LPS-induced IP_3_R2 expression led to Ca^2+^_i_ overload, ER stress, and myocardial pyroptosis while treatment targeting IP_3_R2 and ER stress provided cardioprotective benefits against LPS-induced cardiomyocyte pyroptosis.

### 5.3. IP_3_Rs, Ca^2+^ Signaling, and Ferroptosis (Figure 2C)

Ferroptosis is defined as being iron-dependent and lipid peroxidation-driven PCD [87,101]. The disturbance of intracellular iron and the perturbation of ROS are considered crucial factors in the initiation of ferroptosis [102]. Unlike other types of PCDs, ferroptosis does not require the involvement of traditional death executioner proteins to induce its lethal consequences [103]. Instead, ferroptosis is driven by an imbalance between the accumulation of lipid peroxidation, specifically phospholipid hydroperoxides (PLOOHs) [104], and the insufficiency of endogenous antioxidation systems such as glutathione peroxidase 4 (GPX4), coenzyme Q10 (CoQ10), and tetrahydrobiopterin (BH4) [105,106,107,108]. This imbalance leads to plasma membrane damage and rupture [101,102,103]. Ferroptosis is executed by the accumulation of peroxidized lipids, which leads to the permeabilization of the plasma membrane [107]. In contrast to other types of cell death, ferroptosis has been shown to propagate to neighboring cells [109]. The intercellular propagation of ferroptosis has been proposed to occur through the release of oxidized lipids via exocytic vesicles [107,109]. It has been recognized that ferroptosis plays a significant role in the pathogenesis and progression of numerous cardiovascular diseases including ischemia, cardiac I/R injuries, arrhythmias, sepsis-induced cardiomyopathy, chemotherapy-related cardiotoxicity, iron overload cardiomyopathy, diabetic cardiomyopathy, cardiac hypertrophy, and heart failure [110,111,112]. Among these diseases, mitochondria-dependent ferroptosis has garnered increasing attention due to its pivotal role in subcellular metabolic, redox, and ion homeostasis [113,114]. Alterations in mitochondrial morphology and function commonly predispose cardiomyocytes to ferroptosis [113,114]. Mitochondrial fragmentation, Ca^2+^_Mito_ overload, mtROS overproduction, excessive mitochondrial lipid peroxidation, and disruption of the mitochondrial membrane potential (Δ*Ψ_m_*) have been associated with cardiac ferroptosis and impaired contraction [107,109,110,111,112,113].

Specifically, previous studies have suggested that the sustained elevation of [Ca^2+^]_i_ is linked to the incidence of ferroptosis [115,116,117,118]. Mendoza et al. reported that subtoxic levels of ROS and Ca^2+^ synergistically sensitize Ca^2+^-dependent mPTP opening, resulting in mitochondrial swelling and dysfunction [119]. The dual inhibition of mPTP using cyclosporine A and lipid peroxidation using the selective ferroptosis inhibitor ferrostatin-1 and MitoQ provided protection against I/R injury-mediated mPTP-dependent cell death [119] suggesting the involvement of ferroptosis. Likewise, ferroptosis can also be initiated by intracellular stressors that induce ER stress and Ca^2+^_Mito_ overload mediated by IP_3_Rs [120]. Mitochondria–ER contact sites (MERCS) are specialized subdomains of the ER/SR that physically reside in close apposition to mitochondria [121]. This specific surface enables the local conveyance of Ca^2+^ between IP_3_Rs on the SR/ER membrane and MCU on the mitochondrial membrane, thereby facilitating the Ca^2+^_Mito_ sequestration [2,114]. Mechanistically, the activation of IP_3_Rs causes a significant rise in [Ca^2+^]_Mito_. However, the overwhelming activation of IP_3_Rs can promote electron transport chain (ETC) dysfunction and enhance mtROS production [120]. Consistent with this thought, Pedrera et al. demonstrated a sustained increase in [Ca^2+^]_i_ in NIH-3T3 cells (an embryonic mouse fibroblast cell line) upon treatment with ferroptosis inducers Erastin-1 and RAS-selective lethal 3 (RSL3) [118]. In addition, the occurrence of Ca^2+^_i_ overload and ferroptosis-related phenotypes can be effectively inhibited by the ferroptosis inhibitor Ferrostatin-1 [118]. Other studies have also reported that the rise of [Ca^2+^]_i_ and [Ca^2+^]_Mito_ occurs before the complete lysis of the plasma membrane during ferroptosis [116,122]. Furthermore, IP_3_R-mediated Ca^2+^ release has also been reported to be associated with ferroptosis. In a very recent study by Campos and colleagues, ferroptosis was induced in SH-SY5Y neuroblastoma cells using RSL3, known as an inhibitor of the anti-ferroptosis protein GPX4 [16]. Under RSL3 treatment, they found an increase in ferroptosis, elevated lipid peroxidation products, and a time-dependent overload of Ca^2+^_i_ [16]. Treatment with IP_3_R antagonists either Xestospongin B or carbachol improved cell viability, while carbachol treatment reduced the expression levels of IP_3_R1 under RSL3-induced ferroptosis conditions [16]. Additionally, the knockdown of IP_3_R1 alleviated Ca^2+^_i_ overload and reduced the occurrence of the ferroptosis phenotype in RSL3-treated SH-SY5Y cells [16]. Additionally, Zhou et al. also reported that the administration of the IP_3_R antagonist 2-APB strongly suppressed RSL3-induced ferroptosis in primary rat articular chondrocytes and C28/I2 cells (human chondrocyte cells) [123]. In line with these findings, the anti-ferroptosis effect of IP_3_R antagonists was also observed by Hirata et al. who reported that 2-APB inhibited RSL3-induced ferroptosis in RAW 264.7 cells and HeLa cells [124]. Taken together, the sustained elevation of [Ca^2+^]_i_ reported in association with IP_3_Rs activation can promote ferroptosis in several types of non-cardiac cells. Furthermore, IP_3_R antagonists such as Xestospongin B, carbachol, and 2-APB have been shown to improve cell viability, reduce IP_3_R1 expression, and alleviate Ca^2+^_i_ overload. This indicates the potential role of IP_3_Rs in regulating ferroptosis across various cell types. Further studies are indeed warranted to explore the role of IP_3_Rs and Ca^2+^ in ferroptosis specifically within cardiomyocytes. This research could provide valuable insights into the mechanisms underlying ferroptosis in cardiovascular diseases. A summary of the contribution of IP_3_Rs in different types of programmed cell death is shown in Table 1.

## 6. Conclusions

The intricate involvement of IP_3_Rs in diverse cardiac pathologies and PCD pathways has been progressively explored. The elevation of IP_3_Rs along with the dysregulation of related proteins have been reported in several cardiac pathological conditions including ischemia, I/R injuries, arrhythmias, sepsis-induced cardiomyopathy, cardiac hypertrophy, and heart failure. IP_3_R1 plays a pivotal role in cardiac ischemia and diabetes-induced arrhythmias. On the other hand, IP_3_R2 demonstrates significance in sepsis-induced cardiomyopathy and cardiac hypertrophy. Findings from previous studies also suggest that, upon exposure to various stressors or pathological conditions, there is an initial upregulation of IP_3_Rs which exacerbates Ca^2+^ overload and subsequently activates PCDs. The modulation of IP_3_Rs and their associated proteins, either through genetic interventions or pharmacological approaches, holds therapeutic promise for mitigating Ca^2+^ handling abnormalities and improving cardiac cell survival. Furthermore, IP_3_Rs have been implicated in diverse PCD pathways, including apoptosis, pyroptosis, and ferroptosis emphasizing their multifaceted roles in cardiac pathophysiology. However, there is still a lack of studies investigating the distinct role of IP_3_Rs in other types of PCDs, such as necroptosis and autophagy-dependent cell death. In addition, the potential effects of genetic ablation and pharmacological intervention using IP_3_R antagonists pose promising therapeutic potency against IP_3_R-related pathologies. Understanding the roles of IP_3_Rs provides a foundation for addressing IP_3_R-related pathologies particularly in relation to the initiation of PCD in cardiovascular diseases. Further studies are warranted to gain a comprehensive understanding of the involvement of IP_3_Rs in cardiac pathologies and to unveil their potential implications for future therapeutic interventions.

## Figures and Tables

**Table 1:** A summary of the contribution of IP_3_Rs in different types of programmed cell death (PCDs).

PCDs	PCD-Specific Signaling Pathway Alterations	IP_3_R Signaling		[Ca^2+^] Changes	Pathological Condition /Study Model	Ref.
		In Vitro	In Vivo			
Pyroptosis	↑ NLRP3 ↑ ASC ↑ Caspase 1 ↑ GSDMD-NT ↑ IL-1β ↑ IL-6	↑ IP_3_R1 ↓ ERP44	↑ IP_3_R1 ↓ ERP44	↑ [Ca^2+^]_i_ ↑ [Ca^2+^]_Mito_	in vitro: CMs and H9c2 cells subjected to H/R condition (H: 2 h, R: 24 h) in vivo: SD rats with Cardiac I/R (I: 30 min, R: 120 min)	[13]
	↑ Caspase 1 ↑ GSDMD-NT ↑ IL-1β ↑ IL-6 ↑ TUNEL positive nuclei	↑ IP_3_R2 ↔ IP_3_R1 ↔IP_3_R3	↑ IP_3_R2	↑ [Ca^2+^]_i_	in vitro: NRAMs under LPS stimulation (2 μg/mL for 24 h) in vivo: SD rats with LPS injection (10 mg/kg IP)	[14]
Apoptosis	↑ Bax/Bcl-2	↑ IP_3_R1 ↑ GRP75 ↑ VDAC	N/A	N/A	in vitro: NRAMs subjected to HG stimulation (25 mM for 48 h)	[15]
	↑ Cleaves caspase 9/Caspase 9 ↑ Cleaves caspase3/Caspase 3 ↑ Bax/Bcl-2	↑ IP_3_R1 ↑ GRP75 ↑ VDAC	↑ IP_3_R1 ↑ GRP75 ↑ VDAC	N/A	in vitro: HL-1 cells subjected to HG, TM stimulation (dose not available) in vivo: SD rats with T2DM (STZ 30 mg/kg IV)	[60]
Ferroptosis	↑ Oxidative stress ↑ Lipid peroxidation ↑ Ferroptosis Phenotypes ↔ Cleaves caspase 3	↑ IP_3_R1	N/A	↑ [Ca^2+^]_i_	in vitro: SH-SY5Y cells under RSL3 stimulation (5 μM for 4 h)	[16]

*Abbreviations:* ASC, apoptosis-associated speck-like protein containing a CARD; Bax, BCL2 Associated X; Bcl-2, B-cell leukemia/lymphoma 2; CMs, cardiomyocytes; ERP44, endoplasmic reticulum resident protein 44; GRP75, glucose-regulated protein 75; GSDMD-NT, gasdermin-D *N*-terminus; HG, high glucose; H/R, hypoxia-reoxygenation; IL-1β, interleukin 1β; IL-6, interleukin 6; IP_3_R1, inositol 1,4,5-trisphosphate receptor type 1; IP_3_R2, inositol 1,4,5-trisphosphate receptor type 2; I/R, ischemia–reoxygenation; LPS, lipopolysaccharide; NRAMs, neonatal rat atrial cardiomyocytes; NRCMs, neonatal rat cardiomyocytes; N/A, not applicable; STZ, streptozotocin; TM, tunicamycin; TUNEL, terminal deoxynucleotidyl transferase dUTP nick end labeling; T2DM, type 2 diabetes mellitus; VDAC, voltage-dependent anion channel; [Ca^2+^]_i_, intercellular/cytoplasmic Ca^2+^ (Ca^2+^_i_) concentration; [Ca^2+^]_Mito_, intramitochondrial Ca^2+^ concentration; ↑, upregulation/increased; ↓ downregulation/decreased; ↔, no significant changed.

## List of Abbreviation

AF, atrial fibrillation; ASC, apoptosis-associated speck-like protein containing a CARD; Bax, BCL2 Associated X; Bcl-2, B-cell leukemia/lymphoma 2; CaMKII, Ca^2+^/calmodulin-dependent protein kinase II; CICR, Ca^2+^-induced Ca^2+^ release; CMs, cardiomyocytes; cTnC, cardiac troponin C; ECC, excitation–contraction coupling; ER, endoplasmic reticulum; ERP44, endoplasmic reticulum resident protein 44; GPX4, glutathione peroxidase 4; GRP75, glucose-regulated protein 75; GSDMD-NT, gasdermin-D *N*-terminus; HG, high glucose; H/R, hypoxia-reoxygenation; IL-1β, interleukin 1β; IL-6, interleukin 6; IP_3_, inositol 1,4,5-trisphosphate; IP_3_R1, inositol 1,4,5-trisphosphate receptor type 1; IP_3_R2, inositol 1,4,5-trisphosphate receptor type 2; IP_3_R3, inositol 1,4,5-trisphosphate receptor type 3; I/R, ischemia–reperfusion; LPS, lipopolysaccharide; LTCC, L-type voltage-gated calcium channels; MCU, mitochondrial uniporter; Mfrn, mitoferrin 2; mPTP, mitochondrial permeability transition pore; NCX, sodium–calcium exchanger; NRAMs, neonatal rat atrial myocytes; NRCMs, neonatal rat cardiomyocytes; N/A, not applicable; PCD, programmed cell death; PLN, phospholamban; RyR2, ryanodine receptor type-2; SERCA, sarco/endoplasmic reticulum calcium ATPase; SR, sarcoplasmic reticulum; STZ, streptozotocin; TLRs, toll-like receptors; TM, tunicamycin; TNFR1, tumor necrosis factor receptor 1; TNF-α, tumor necrosis factor-α; TUNEL, terminal deoxynucleotidyl transferase dUTP nick end labeling; T2DM, type 2 diabetes mellitus; VDAC, voltage-dependent anion channel; [Ca^2+^], Ca^2+^ concentration; [Ca^2+^]_i_, intercellular/cytoplasmic Ca^2+^ (Ca^2+^_i_) concentration; [Ca^2+^]_Mito_, intramitochondrial Ca^2+^ concentration; mitochondrial membrane potential (Δ*Ψ_m_*).
